# Injectable organic-inorganic hybrid hydrogels for bone defect repair

**DOI:** 10.3389/fbioe.2025.1563546

**Published:** 2025-03-18

**Authors:** Huan Zhang, Shuo Ding, Huai Xue, Shuguang Wang, Xiaoyu Quan, Dong Zhang, Xiao Liu, Hai Tang

**Affiliations:** ^1^ Department of Orthopaedics, Beijing Friendship Hospital, Capital Medical University, Beijing, China; ^2^ School of Second Clinical Medicine, Xuzhou Medical University, Xuzhou, Jiangsu, China; ^3^ Department of Trauma Center, The Affiliated Hospital of Xuzhou Medical University, Xuzhou, Jiangsu, China; ^4^ Department of Emergency Medicine, The Affiliated Hospital of Xuzhou Medical University, Xuzhou, Jiangsu, China; ^5^ Department of Emergency, Fengxian People’s Hospital, Xuzhou, Jiangsu, China; ^6^ The Wallace H. Coulter Department of Biomedical Engineering, Georgia Institute of Technology and Emory University, Atlanta, GA, United States

**Keywords:** biomacromolecules, injectable hydrogel, hybrid hydrogel, bone defect, GelMA

## Abstract

Bone defects caused by trauma, tumor resection, and surgery present significant clinical challenges, often resulting in complications such as delayed union, nonunion, and even long-term functional impairment. Current treatments, including autografts and allografts, are limited by donor site morbidity, immune rejection, and pathogen transmission, highlighting the need for developing reliable synthetic alternatives. To address these challenges, we report a binary composite hydrogel combining gelatin methacryloyl (GelMA) and κ-Carrageenan, reinforced with calcium phosphate cements (CPC). GelMA ensures rapid gelation and biocompatibility, κ-carrageenan improves injectability, and CPC enhances mechanical strength and osteogenic activity, collectively creating a robust and versatile hydrogel system. Furthermore, the hydrogel’s injectable, adaptive, and self-healing characteristics enable it to conform to irregular bone defect sites, providing mechanical support and osteogenic stimulation. It also releases bioactive components to accelerate bone regeneration. With exceptional toughness and resilience, this hydrogel recovers its shape after deformation, positioning it as a promising candidate for clinical bone defect repair applications.

## Introduction

Bone defects caused by trauma, tumor resection, and surgical procedures represent significant clinical challenges with high associated morbidity (Che et al., 2019; Collon et al., 2021; Wang R. et al., 2024). Severe bone defects can lead to complications such as delayed union, nonunion, and impaired function, often resulting in permanent disability (Balogh et al., 2012; Dong et al., 2023). Current strategies for the treatment of bone defects primarily involve autografts and allografts. However, these methods are not without limitations. Autografts, while effective, are associated with donor site morbidity, including pain, blood loss, infection, and disruption of the local anatomical structure, potentially hindering functional recovery (Piacentini et al., 2019). Additionally, the limited availability of autograft tissue and the finite amount of bone that can be harvested pose practical challenges. Allografts, while able to overcome certain issues, are prone to triggering immune rejection and carry the risk of pathogen transmission. Furthermore, allogeneic bone lacks viable cells, resulting in insufficient osteogenic capacity, and the processing procedures may reduce its mechanical strength, further increasing the complexity of its use. (Rougier et al., 2022; Sharifi et al., 2022; Wang F. et al., 2025). Some existing synthetic materials may produce degradation byproducts after implantation that could cause toxicity or irritation to surrounding tissues and cells, hinder healing, and are associated with high costs and challenges in large-scale production (Yu et al., 2024). These limitations have spurred the development of synthetic bone substitutes as an alternative (Pitacco et al., 2023; Matsuura et al., 2024).

Over the past few decades, significant progress has been achieved in the field of bone tissue engineering, with materials such as hydroxyapatite and calcium phosphate cements (CPC) being widely used in clinical applications (Dapporto et al., 2022). However, despite their clinical success, CPC-based materials still face significant challenges. Once hardened, CPC exhibits low toughness, which limits its applicability in load-bearing commonly encountered in orthopedic surgery (Wang et al., 2021; Zhao et al., 2025). Thus, there remains a critical need to improve the mechanical properties of CPC and other synthetic bone substitutes to enhance their performance in bone defect repair.

Hydrogels, characterized by their crosslinked polymer networks and high water content, have emerged as a promising solution for bone regeneration due to their tunable mechanical properties, biocompatibility, and adaptability to irregular defect geometries (Sun et al., 2012; Zhang et al., 2021; Ni et al., 2022; Wang et al., 2022). However, conventional hydrogels often require high monomer concentrations or extensive post-processing to achieve desirable mechanical properties, which can result in slow gelation, excessive by-product formation, and poor control over the gelation process (Seyedsalehi et al., 2024). While organic-inorganic composite hydrogels incorporating materials such as clays (Lin et al., 2010), graphene (Shao et al., 2014), and MXene (Wang W. et al., 2024) have demonstrated improved mechanical performance, concerns regarding toxicity and long-term biocompatibility persist. Therefore, the development of injectable, moldable, and biologically safe hydrogels remains a critical challenge in bone tissue engineering.

Injectable hydrogel formulations, in particular, offer the advantage of minimally invasive delivery and precise defect filling (Jin et al., 2023; Tang et al., 2024). A key limitation of many hydrogel precursor solutions is their low viscosity, which complicates injection and molding processes (Zuo et al., 2023; Wang H. et al., 2025). Additionally, residual monomers from incomplete polymerization can induce cytotoxicity, necessitating extensive post-treatment such as dialysis. Commonly used biopolymers, including sodium alginate and chitosan, often require crosslinking agents for gelation and are highly sensitive to environmental factors such as pH, limiting their reproducibility and functionality. Gelatin methacrylate (GelMA), a widely used material in tissue engineering, offers rapid photoinitiated crosslinking and excellent biocompatibility, making it particularly suitable for soft tissue repair (Wang Y. et al., 2023). Its thermoresponsive behavior, where viscosity increases with decreasing temperature, facilitates injectability (Che et al., 2023). However, maintaining a low-temperature environment during clinical procedures is often impractical, restricting its operational feasibility. Κ-Carrageenan, a natural polysaccharide derived from red algae, has gained attention for its biocompatibility, thickening properties, and ability to form gels in the presence of monovalent ions (Krebs et al., 2023; Jiang et al., 2024). While its interaction with divalent ions is weaker, κ-carrageenan’s unique properties make it an attractive component for hydrogel design (Lei et al., 2022; Wang F. F. et al., 2023).

In this study, we developed a dual-network hydrogel system combining GelMA and κ-carrageenan. GelMA provides rapid gelation and a nutrient-rich matrix, while κ-carrageenan enhances the thixotropic behavior of the precursor solution, enabling injectability and ease of molding. To further improve mechanical performance, calcium phosphate cement (CPC) was incorporated, creating an organic-inorganic composite hydrogel with enhanced structural integrity and osteogenic potential.

The injectable hydrogel adaptively fills bone defects, combining mechanical reinforcement and osteogenic stimulation in a single bioactive system. By leveraging the synergistic properties of GelMA, κ-carrageenan, and CPC, this novel formulation addresses key limitations of existing synthetic bone repair materials. Its room-temperature injectability and moldability offer significant potential for bone tissue engineering and orthopedic applications, paving the way for more effective clinical treatments for bone defects.

## Results and discussion

**FIGURE 1:**
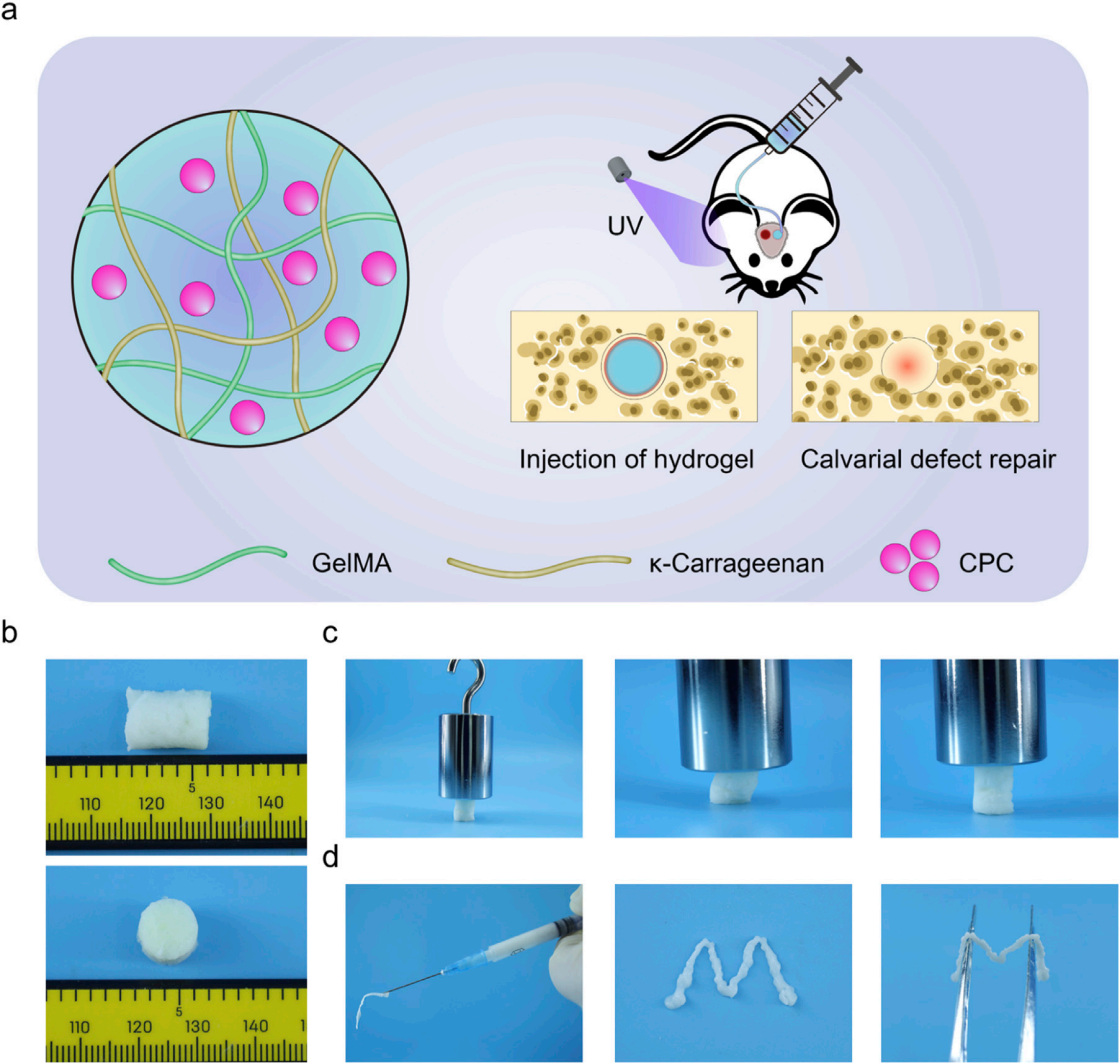
**(a)** Schematic illustration showing the preparation of injectable CPC hydrogel and its application in bone regeneration. **(b)** Optical image showing the cylindrical gel prepared through mold casting followed by photo-initiated crosslinking, exhibiting a uniform morphology. **(c)** Image demonstrating the GKP hydrogel’s ability to withstand a 500 g weight, showing deformation under pressure without fracture. **(d)** Illustration of the injectability and *in situ* molding ability of this hybrid hydrogel system. The precursor was injected to form an “M” shape, and upon gelation, it retained the molded structure.

To overcome the challenges associated with conventional injectable hydrogels, including prolonged gelation times and inadequate mechanical strength, this study developed an innovative injectable bioactive hydrogel system featuring rapid gelation and structural stability, as depicted in Figure 1A. The hydrogel precursor solution was formulated using GelMA and κ-carrageenan, which synergistically contribute to structural integrity and biocompatibility. To further enhance osteogenic potential, an inorganic bioactive component was integrated, creating an organic-inorganic composite system. The hydrogel undergoes rapid gelation upon photo-crosslinking. This property is especially beneficial for bone defect repair, as it enables injectable and adaptive filling, ensuring *in situ* gelation, providing mechanical support, and promoting osteogenic stimulation.

Figure 1B illustrates the fabrication of cylindrical GelMA-κ-carrageenan/CPC (GKP) hydrogels using a mold, highlighting their uniform morphology and well-defined structure. To assess the mechanical properties, a 500-g weight was applied to the hydrogel, resulting in less than 5% deformation. This confirmed the hydrogel’s high modulus and suitability for load-bearing applications (Figure 1C). Additionally, the hydrogel exhibited remarkable toughness and shape recovery, as it returned to its original form after deformation upon force removal. Figure 1D illustrates the injectability and rapid shape retention of the precursor solution. By extruding the solution through a syringe to form the letter “M” and subsequently photo-crosslinking it, the hydrogel maintained its precise shape, which could be lifted with tweezers, demonstrating exceptional shape fidelity. These properties, combined with the material’s biocompatibility and injectability, position it as a promising candidate for bone regeneration applications.

**FIGURE 2:**
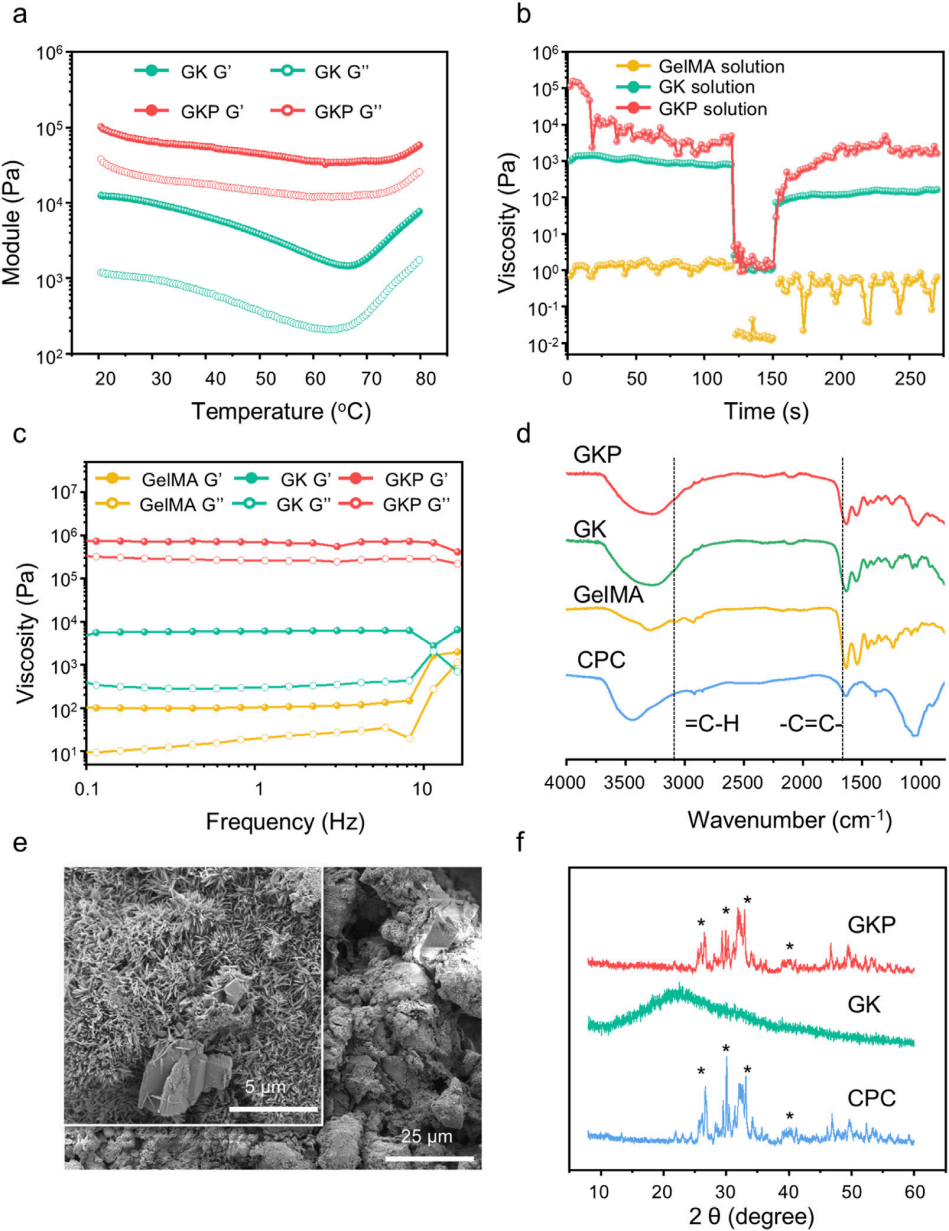
Materials characteristic of precursor solutions and hydrogels. **(a)** Modulus variation of precursor solutions at different temperatures. **(b)** Rheological measurements to simulate the shear-thinning and recovery behaviors of different precursors with various concentrations: Step I, at a shear rate of 0.1 s^−1^, Step II, at a shear rate of 100 s^−1^, and Step III, at a shear rate of 0.1 s^−1^ **(c)** Rheological behavior of hydrogels under dynamic frequency sweep tests. **(d)** FTIR spectra of CPC, GelMA, GK, and GKP gels. **(e)** SEM images of GKP hydrogels. **(f)** XRD patterns of CPC and bioactive hydrogels, with characteristic peaks marked by an asterisk (*).

The processability of various precursor solutions was evaluated using a rotational rheometer, beginning with a temperature sweep test to determine the processing window. As illustrated in Figure 2A and Supplementary Figure S1, the inclusion of κ-carrageenan markedly enhanced the structural stability of the precursor solutions. In contrast to the pure GelMA solution, which displayed lower moduli across the 20°C–80°C range (with the loss modulus surpassing the storage modulus), the material exhibited predominantly viscous behavior. For GelMA, the modulus initially decreased between 20°C–45°C, a phenomenon attributed to its thermosensitive properties, which reduce viscosity as temperature rises. Subsequently, the modulus increased, likely due to partial polymerization triggered by the initiator at higher temperatures. A comparable trend was observed in the GelMA-κ-carrageenan (GK) precursor solution, although this effect was less pronounced in the GKP precursor solution. Notably, both the GK and GKP precursor solutions demonstrated storage moduli consistently higher than their loss moduli across a wider temperature range, indicating superior static molding capability. The incorporation of CPC inorganic particles further augmented the modulus of the precursor solutions, enhancing their plasticity and structural integrity.

The thixotropic properties of the precursor solutions were investigated under rotational mode at room temperature to assess their printability (Figure 2B). The test protocol comprised three stages: (i) application of a shear rate of 0.1 s^−1^ to simulate static conditions, (ii) application of a shear rate of 100 s^−1^ to mimic the shear forces encountered during hydrogel extrusion, and (iii) reduction of the shear rate to evaluate viscosity recovery. The pure GelMA solution exhibited consistently low viscosity, making it excessively fluid to retain shape at room temperature. In contrast, the GK and GKP precursor solutions displayed pronounced thixotropic behavior, characterized by flowability under high shear rates and shape retention under low shear conditions, thereby enabling injectability and printability at ambient temperature. Notably, the GKP precursor solution, enhanced by the incorporation of inorganic particles, achieved higher viscosity under low shear due to particle interactions while maintaining shear-thinning behavior comparable to GK under high shear, demonstrating superior rheological performance. Further characterization of the resultant hydrogels was conducted using frequency sweep measurements (Figure 2C). Across both low and high frequencies, the storage modulus (G’) consistently exceeded the loss modulus (G’’), with the corresponding curves exhibiting parallelism. This behavior indicates the formation of a uniform, intact gel network structure with robust resistance to disruption, underscoring the material’s structural integrity and stability.

The chemical structures of the precursors and hydrogels were characterized using Fourier-transform infrared (FTIR) spectroscopy. As depicted in Figure 2D, the absorption peaks in the range of 3,300–3,000 cm^−1^ correspond to unsaturated C-H vibrations, indicative of the -C=C- bonds in GelMA. Following photo-initiated crosslinking for 2 min, the peaks associated with double bonds and unsaturated C-H vibrations in both the GK and GKP groups disappeared, confirming the occurrence of *in situ* polymerization in these systems. Complete polymerization of hydrogel precursors is critical, as it typically results in reduced cytotoxicity compared to unreacted monomers, a vital consideration for clinical applications. The characteristic peaks of CPC were observed at 3,500 and 1,050 cm^−1^, partially overlapping with peaks from other components. Notably, the peak shifts in GKP at these positions were more pronounced than in GK, suggesting successful incorporation of inorganic CPC and the formation of a composite bioactive hydrogel. The microstructures of pure CPC scaffolds, GK hydrogels, and GKP hydrogels were examined using scanning electron microscopy (SEM) (Figure 2E; Supplementary Figure S2). The pure CPC scaffold exhibited large pores and a loosely structured inorganic network. In contrast, the GK hydrogel displayed a denser network, contributing to enhanced mechanical toughness. The GKP hydrogel demonstrated an even denser structure compared to the pure CPC scaffold, facilitating a more uniform dispersion of CPC within the hydrogel matrix. Elemental analysis of the GKP hydrogel surface via energy-dispersive spectroscopy (EDS) (Supplementary Figure S3) revealed a homogeneous distribution of calcium (Ca) and phosphorus (P) elements, confirming the uniform incorporation of bioactive particles. Additionally, X-ray diffraction (XRD) analysis (Figure 2F) identified the phase composition of the GKP hydrogel, which included tetracalcium phosphate (TTCP, Ca_4_(PO_4_) _2_O) and dicalcium phosphate anhydrous (DCPA, CaHPO_4_), precursors for hydroxyapatite (HAp, Ca_10_(PO_4_) _6_(OH)_2_). In comparison, the XRD pattern of the purely organic GK material exhibited a broad diffuse peak. The intensities of HAp and TTCP peaks in GKP were significantly lower than those in CPC, likely due to the shielding effect of the organic matrix.

Bone tissue, as a hard tissue, necessitates filling materials with appropriate mechanical properties for defect repair. In this study, the GKP material demonstrated excellent molding capability and active material-loading capacity. Compression tests were conducted to evaluate whether the organic-inorganic composite system could enhance the mechanical performance of clinical CPC scaffolds (Supplementary Figure S4a). Clinically used CPC scaffolds are brittle, with compressibility typically below 10%, representing a significant limitation. Conversely, GelMA, due to its low mechanical strength, is primarily employed for soft tissue regeneration. The introduction of κ-carrageenan alone resulted in a modulus lower than that of single-component gels, likely because κ-carrageenan cannot crosslink in aqueous solutions at room temperature and only maintains shape. In the GK system, the dual components failed to establish strong interactions, and the gel network was disrupted under compression, leading to reduced compressive resistance. However, the incorporation of CPC facilitated robust organic-inorganic composite interactions, as evidenced by the increased initial modulus. During the initial compression stage, although the GKP curve was positioned below that of the pure CPC scaffold, the GKP material exhibited compressive deformation exceeding 10%. Remarkably, GKP sustained increasing compressive strength up to 3.11 MPa, demonstrating superior compressive properties. The compressive modulus, calculated from the elastic stage of the curves (Supplementary Figure S4b), revealed that the Young’s modulus of the GKP hydrogel was significantly higher than that of GelMA and GK hydrogels, reaching 79% of the CPC scaffold’s modulus. These results underscore the excellent mechanical performance of GKP, highlighting its potential as a supporting material for bone defect repair.

The swelling behavior of the hydrogels in simulated body fluid (SBF), as calculated using Equations 1, is shown in Supplementary Figure S5. Due to the hydrophilic nature of GelMA and κ-carrageenan, their swelling ratios exceeded 100% of their original weight, indicating that purely organic networks are insufficient for withstanding humid environments. The introduction of CPC inorganic components resulted in the formation of an organic-inorganic composite hydrogel network, significantly reducing the swelling ratio to 81%, lower than that of the GelMA and GK groups. All hydrogels exhibited degradability in the presence of collagenase, as evidenced by the weight retention profiles shown in Supplementary Figure S6, which were quantitatively analyzed using Equation 2. Pure hydrogels degraded too rapidly to effectively fill defects, whereas the GK hydrogel exhibited slower degradation due to the presence of the κ-carrageenan macromolecular network. This increased network density and enhanced component interactions improved system stability. The organic-inorganic hybrid GKP hydrogel exhibited an even slower degradation rate, requiring an extended period for complete degradation. This prolonged degradation behavior enhances the bioactivity of the CPC-hydrogel scaffold, enabling sustained CPC release rather than rapid disintegration.

### Evaluation of biocompatibility and osteogenic effects of hybrid hydrogels

**FIGURE 3:**
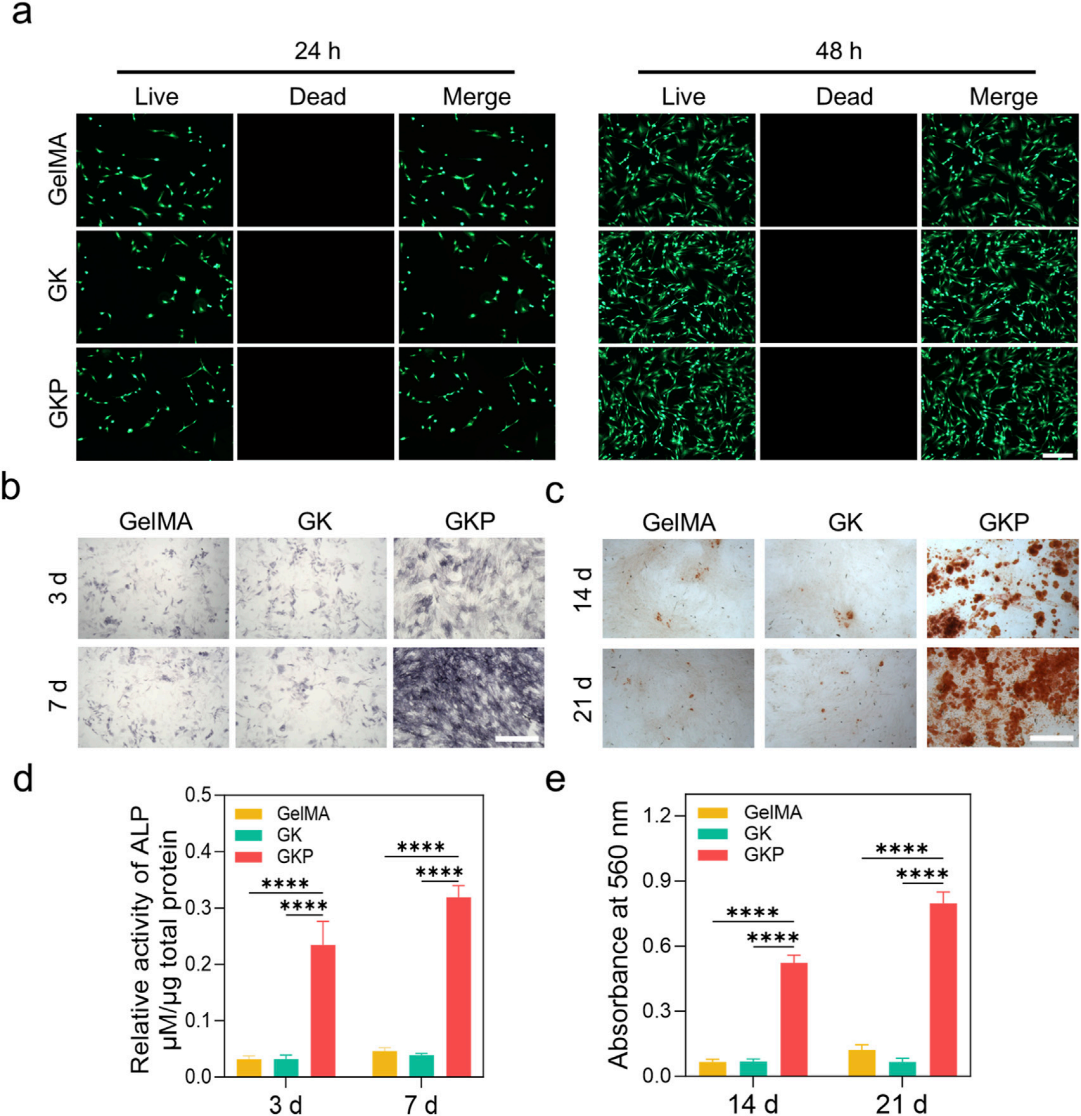
Biocompatibility and osteogenic differentiation evaluation of GelMA, GK, and GKP hydrogels. **(a)** Live/Dead staining images of cells encapsulated in GelMA, GK, and GKP hydrogels at 24 and 48 h, showing live (green) and dead (red) cells. **(b)** ALP staining of cells at 3 and 7 days, indicating ALP activity. **(c)** ARS staining at 14 and 21 days, demonstrating mineralized matrix deposition. **(d)** Quantitative analysis of relative ALP activity at 3 and 7 days. **(e)** Quantification of ARS absorbance at 560 nm at 14 and 21 days. Data are presented as mean ± SD, with statistical significance indicated. Scale bars: 100 μm. (n = 3; *p < 0.05, **p < 0.01, ***p < 0.001, ****p < 0.0001).

Regularly shaped GelMA, GK, and GKP hydrogels were fabricated using molds and co-cultured with bone marrow mesenchymal stem cells (BMSCs) for 1 and 3 days. Cell viability and proliferation were assessed quantitatively across different hydrogel groups using the Cell Counting Kit-8 (CCK-8) assay. The results indicated that all hydrogel formulations supported BMSC proliferation, with no significant differences in optical density (OD) values observed at the same time points (Supplementary Figure S7). These findings suggest that neither the precursor components of the gel nor the GKP hydrogel exerted any detrimental effects on cell proliferation. Furthermore, live/dead cell staining was performed at 24 and 48 h to evaluate cellular growth. The data revealed a significant increase in the number of viable BMSCs over the culture period, with no apparent cytotoxicity, confirming that the materials effectively support cell growth (Figure 3A). To further assess the osteogenic potential of the hydrogels, alkaline phosphatase (ALP) staining and Alizarin Red S (ARS) staining were conducted. During osteogenic differentiation, BMSCs secrete substantial amounts of alkaline phosphatase. Figure 3B illustrates the ALP staining results of BMSCs cultured in conditioned media from different hydrogels, with only the GKP group exhibiting marked ALP secretion at both 3 and 7 days. As osteoblasts mature, they deposit calcium salts, forming mineralized nodules. Figure 3C shows ARS staining of BMSCs cultured in the conditioned media, highlighting significant calcium salt deposition in the GKP group, indicative of extensive osteoblast formation. The quantitative analyses of ALP and ARS staining further substantiate this observation (Figures 3D, E). In conclusion, the GKP hydrogel significantly enhanced the osteogenic differentiation of BMSCs.

### Immunofluorescence analysis and osteogenic differentiation performance

**FIGURE 4:**
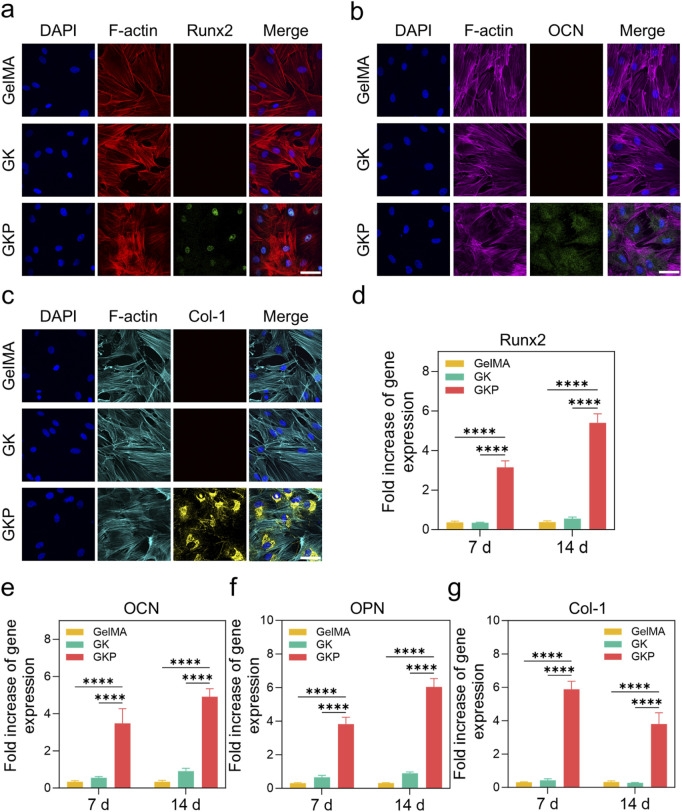
Effect of three hydrogel extracts (GelMA, GK, GKP) on the osteogenic differentiation of BMSCs. **(a–c)** Confocal microscopy images showing the expression of osteogenic markers in BMSCs (Runx2, OCN, Col-1) after 7 days or 14 days of treatment. Scale bar: 200 μm. **(d–g)** RT-qPCR analysis showing the expression levels of osteogenesis-related genes (Runx2, OCN, OPN, Col-1) in BMSCs treated with the hydrogel extracts for 7 or 14 days. All data are presented as mean ± SD (n = 3; *p < 0.05, **p < 0.01, ***p < 0.001, ****p < 0.0001).

Immunofluorescence staining was performed to evaluate the protein expression levels of osteogenic markers in BMSCs cultured with different hydrogels (Figures 4A–C). The expression of Runt-related transcription factor 2 (Runx2), osteocalcin (OCN), and type I collagen (Col-1) were assessed to determine the osteogenic potential of the materials. In both the GelMA and GK groups, no significant protein fluorescence signals were detected, indicating that these precursor hydrogels did not promote BMSC differentiation into osteoblasts. In contrast, the GKP group exhibited prominent fluorescence signals, indicating the formation of a large number of osteoblasts and confirming the superior osteogenic performance of the GKP hydrogel. The osteogenic differentiation capability of the materials is crucial for bone regeneration. To further investigate this, BMSCs were cultured in hydrogel extracts, and the mRNA expression levels of osteogenesis-related genes were quantified using real-time PCR (RT-qPCR) after 7 and 14 days of culture. As shown in Figures 4D–G, the expression levels of osteogenic genes, including Runx2, OCN, Col-1, and osteopontin (OPN), increased over time. Runx2, an early osteogenic marker, exhibited high expression at the early stages but decreased gradually with extended culture time. In contrast, OCN, OPN, and Col-1, which are late-stage osteogenic markers, showed sustained increases in expression. Compared to the GelMA and GK groups, the GKP group demonstrated significant upregulation of osteogenic markers at both 7 and 14 days. These results indicate that the incorporation of CPC effectively enhanced bone regeneration and highlighted the efficient release capacity of the GKP hydrogel.

### *In vivo* evaluation of bone-tissue regeneration with cranial defect models

**FIGURE 5:**
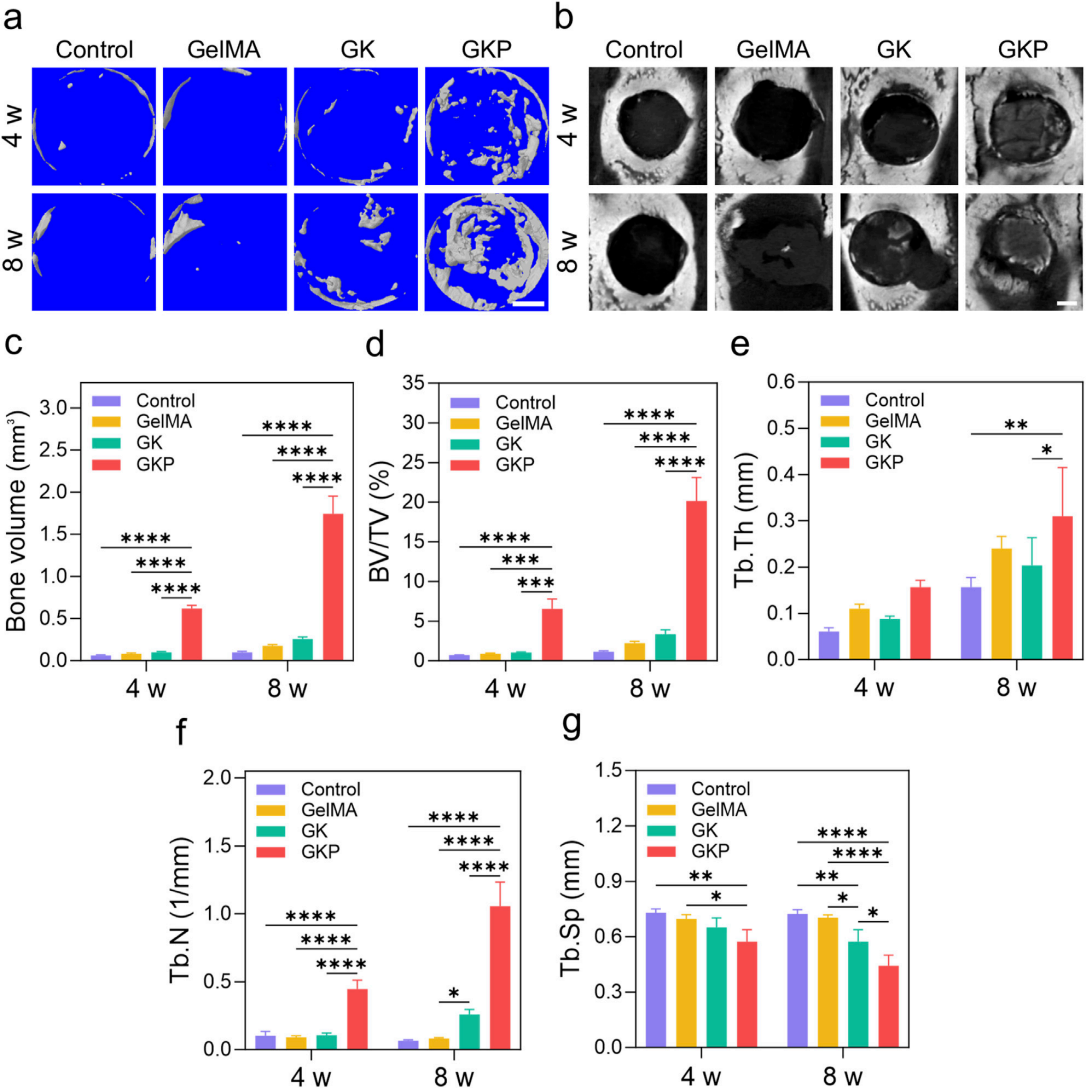
Micro-CT analysis and bone microstructural parameters of cranial defects at 4 and 8 weeks. **(a)** Representative 3D reconstructed micro-CT images of bone regeneration in the defect area for control, GelMA, GK, and GKP groups. Scale bar: 1 mm **(b)** 2D micro-CT images showing the cross-sectional view of the defect sites. Scale bar: 1 mm. **(c–g)** Quantitative analysis of micro-CT parameters, including **(c)** BV, **(d)** BV/TV, **(e)** Tb.Th, **(f)** Tb.N, and **(g)** Tb. Sp at 4- and 8-week post-treatment. Data are presented as mean ± SD, with statistical significance indicated. *p < 0.05, **p < 0.01, ***p < 0.001, ****p < 0.0001.

The excellent reliability and biological functionality of GKP scaffolds warrant further investigation into their potential for bone tissue regeneration. The scaffolds align well with the cranial defect during surgery, and no instances of infection or mortality were observed in any group at 4 or 8 weeks. Tissue morphogenesis and the development of the mineralized matrix at the defect site were evaluated using both 2D and 3D analysis (Figures 5A, B). In the control group, the defect remains a large cavity after 4 weeks, indicating that the rat was unable to self-repair the cranial defect. In the GelMA and GK groups, bone formation remains incomplete. Notably, mineralized bone deposition is observed along the alignment direction of the scaffold struts in the GKP group, suggesting that new bone can grow along the scaffolds due to the incorporation of an inorganic osteogenic bioactive component. This forms an organic-inorganic hybrid bioactive scaffold. Micro-architectural parameters of the newly formed bone within the cranial defect further support these observations (Figures 5C–G). The bone volume (BV) in the GelMA group (0.18 ± 0.02 mm^3^ at 8 weeks) and the GK group (0.26 ± 0.03 mm^3^ at 8 weeks) are greater than that of the control group (0.10 ± 0.01 mm^3^), while the GKP group (1.74 ± 0.21 mm^3^) demonstrates the largest volume of bone regenerative tissue (Figure 5C). Similarly, the bone tissue volume per total tissue volume (BV/TV) in the GKP group (Figure 5D, 6.56% ± 1.22% at 4 weeks and 20.14% ± 2.97% at 8 weeks) is significantly higher than in the GelMA group (0.90% ± 0.08% and 2.24% ± 0.23% at 4 and 8 weeks, respectively), the GK group (1.03% ± 0.09% and 3.34% ± 0.58% at 4 and 8 weeks, respectively), and the control group (0.74% ± 0.02% and 1.17% ± 0.10% at 4 and 8 weeks). The trabecular thickness (Tb. Th, Figure 5E) and trabecular number (Tb. N, Figure 5F) exhibit similar trends. Additionally, trabecular separation (Tb. Sp, Figure 5G) is smallest in the GKP group, suggesting the densest new bone tissue formation. These results collectively demonstrate the outstanding osteogenic performance of the GKP group.

**FIGURE 6:**
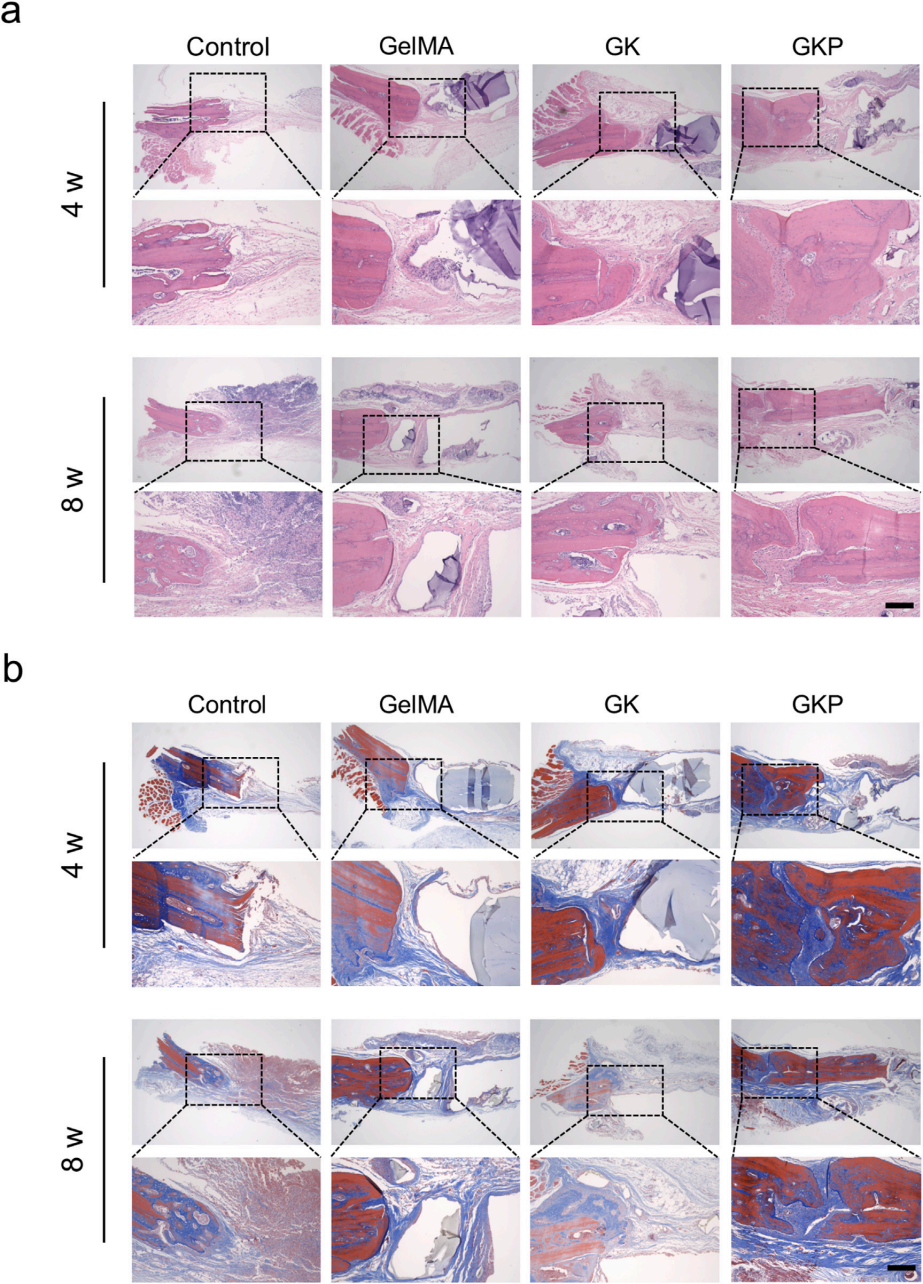
Histological analysis of bone regeneration using H&E and MT staining at 4 and 8 weeks. **(a)** HE staining reveals the tissue morphology within the bone defect site in control, GelMA, GK, and GKP groups. Scale bar: 200 μm. **(b)** MT staining illustrates the collagen fiber distribution within the defect regions. Blue regions represent immature collagen, and red regions indicate mature collagen. Scale bar: 200 µm.

Next, the scaffolds were sectioned, and histological staining was performed to evaluate tissue characteristics. Hematoxylin-eosin (H&E) and Masson’s trichrome (MT) staining results are presented in Figures 6A, B. Figure 6A shows the H&E staining of specimens at 4- and 8-week post-implantation. In the control group, the defect area is filled with minimal loose tissue at 4 weeks, and by 8 weeks, a small amount of newly formed bone matrix and loose connective tissue is present in the defect region. In the GelMA and GK scaffold groups, the defect sites are filled with loose tissue and few blood vessels. In contrast, the GKP group shows the most extensive mature, dense bone tissue. These results suggest that organic-inorganic hybrid bioactive scaffolds can effectively stimulate bone repair. MT staining, which specifically stains collagen fibers, was used to evaluate the formation and maturation of bone tissue (Figure 6B). In the control group, the defect sites are filled with loose connective tissue, and no significant collagen fiber formation is observed. In the GelMA and GK groups, small amounts of immature collagen fibers (blue regions) are visible. However, the GKP group exhibits the largest area of robust mature collagen fibers (red regions) at 8 weeks. These findings confirm that the GKP scaffolds demonstrate strong osteogenic potential.

**FIGURE 7:**
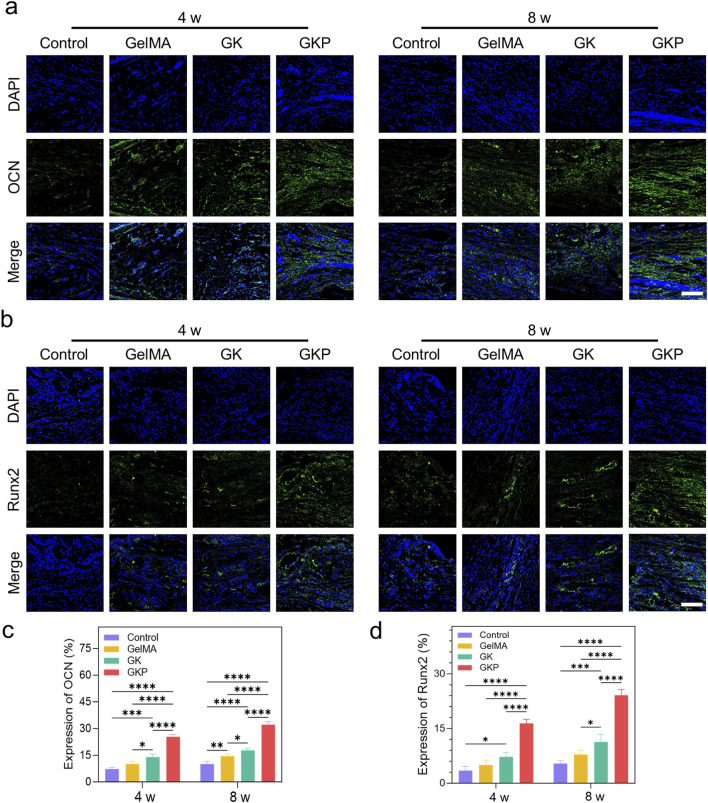
Immunofluorescence staining and quantitative analysis of osteogenic marker expression (OCN and Runx2) in different hydrogel groups after 4 and 8 weeks of implantation. **(a)** Immunofluorescence images showing the expression of OCN in the control, GelMA, GK, and GKP groups at 4 and 8 weeks. Nuclei are stained with DAPI (blue), and OCN expression is marked in green. **(b)** Immunofluorescence images showing the expression of Runx2 in the different groups at 4 and 8 weeks. Runx2 ex-pression is marked in green. Scale bar: 100 μm. **(c–d)** Quantitative analysis of fluorescence intensity for OCN **(c)** and Runx2 **(d)**. (*p < 0.05, **p < 0.01, ***p < 0.001, ****p < 0.0001). Data are presented as mean ± SD (n = 3).

Immunofluorescence staining was employed to further evaluate the effect of the scaffolds on bone regeneration. The results revealed the most intense expression of OCN (Figure 7A) and Runx2 (Figure 7B) in the GKP group. OCN, a secreted acidic protein, is a key osteogenic marker that indicates new bone formation (Song et al., 2022). Runx2, a critical molecular hub integrating Wnt and bone morphogenetic protein (BMP) signaling, plays an essential role in osteoblast differentiation (Wei et al., 2015). Notably, the expression levels of osteoblast markers in the control group were significantly lower compared to those observed in the GelMA and GK groups. OCN and Runx2 expression were most pronounced in the GKP group, indicating enhanced bone tissue formation. Quantitative analysis of fluorescence intensity revealed that, at 8 weeks post-implantation, the relative fluorescence intensity of OCN in the control, GelMA, GK, and GKP groups was 10.07% ± 1.51%, 14.47% ± 1.24%, 17.75% ± 1.08%, and 32.21% ± 1.61%, respectively (Figure 7C). The expression trend of Runx2 mirrored that of OCN (Figure 7D), with relative fluorescence intensities in the control, GelMA, GK, and GKP groups at 8 weeks being 5.38% ± 0.81%, 7.88% ± 1.15%, 11.29% ± 2.13%, and 24.13% ± 1.56%, respectively. In summary, the GKP group, which contains bioactive CPC, showed the most extensive positive staining for OCN and Runx2, underscoring the superior osteogenic potential of these scaffolds.

The evaluation of micro-CT and histological staining demonstrates consistent osteogenic capabilities. The organic-inorganic hybrid bioactive scaffolds, with their high strength, provide an appropriate modulus for bone regeneration. The bioactive inorganic component creates a favorable microenvironment for bone repair. Furthermore, the GKP group appears to promote osteogenic differentiation of BMSCs *in vitro* and enhances bone regeneration *in vivo*. Clinically, bone defect shapes are often variable, and the ability of GKP scaffolds to adapt to different defect sites offers significant advantages for bone defect repair. Their injectable and adaptive properties are crucial for *in situ* gelation, providing mechanical support and osteogenic stimulation. The scaffold releases osteogenic bioactive components to accelerate bone regeneration. Furthermore, its adaptive properties accommodate early patient mobilization, which redistributes mechanical loads and allows the material to regain its structure, restoring support at the defect site. Upon the application of further force, the hydrogel deforms but recovers its original shape once the pressure is released, demonstrating excellent toughness and resilience. These mechanical properties, coupled with the material’s biocompatibility and injectability, underscore its potential as a promising candidate for bone defect repair.

### Biocompatibility evaluation of the GKP hydrogel *in vivo*

During the final euthanasia of the rats at 8 weeks, we collected the heart, lungs, liver, kidneys, and spleen from the experimental group, as well as the corresponding organs from healthy rats. Histological examination using HE staining revealed that, compared to the healthy rats, the organs from the experimental group showed no significant signs of inflammatory cell infiltration, necrosis, or other pathological changes (Supplementary Figure S8). This indicates that the GKP hydrogel is safe for use *in vivo*.

## Conclusion

In this study, GKP hydrogel was synthesized through a one-pot process. Owing to its rapid cross-linking and injectable properties, it can be easily molded to fit *in situ* defects. The GelMA-κ-carrageenan network provided a strong and resilient structure, while the inorganic CPC phase enhanced bioactivity, facilitating bone regeneration. The GKP hydrogel demonstrated excellent biocompatibility and supported cell proliferation. Furthermore, the efficient retention of CPC within the hydrogel system promoted the mRNA expression of key osteogenic genes, including Runx2, OCN, OPN, and Col-1. The enhanced osteogenic activity of GKP was attributed to the increased CPC content and the stability of the hydrogel system. This study provides insights into the synthesis of injectable hydrogels, suggesting that the biological properties of CPC may be further optimized through controlled synthesis to enhance their osteogenic potential. Although the cranial defect model in rats has been validated in research, further verification of its safety and efficacy in larger animal models is lacking. Additionally, the study primarily assessed the bone regeneration capability of the material over the short term (4 weeks and 8 weeks), but it lacks long-term data to demonstrate its degradation behavior and long-term mechanical performance. In future studies, it will be necessary to further validate the osteogenic performance of GKP hydrogel in larger animals such as rabbits and pigs, as well as conduct specific preclinical safety and efficacy studies to advance the material toward practical application.

## Materials and methods

### Chemicals and reagents

GelMA, κ-carrageenan and Lithium Phenyl (2,4,6-trimethylbenzoyl) phosphinate (LAP, 98%) were purchased from Aladdin Reagent Inc. (Shanghai). Tetracalcium phosphate (TTCP, Ca_4_(PO_4_)_2_O) and dicalcium phosphate anhydrous (DCPA, CaHPO4, 98%) were supplied by Macklin Biochemical Co., Ltd. (Shanghai, China). CPC powder was prepared by equimolar mixing of TTCP and DCPA. All reagents were used as received without further purification. In this experiment, all purified water was obtained from a Millipore system with an electronic conductance of 18.2 MΩ cm.

### Preparation of GKP hydrogels

The hybrid hydrogel was fabricated using a one-step polymerization method. Specifically, a predetermined amount of GelMA and κ-carrageenan were dissolved in purified water under stirring at 60°C to prepare a 10 wt% GelMA and 2 wt% κ-carrageenan solution. Subsequently, 0.1 wt% LAP photoinitiator and 10 wt% CPC were added to the solution and dispersed via ultrasonic treatment. The resulting pre-solution was transferred into a syringe and injected into a pre-designed template. The hybrid hydrogel was formed by exposing the solution to UV light (365 nm, 36 W) for 2 min and was designated as GKP Hydrogels. Hydrogels without CPC were defined as GK, while a 10 wt% GelMA hydrogel served as a hydrogel control group. Additionally, a clinically used CPC scaffold was included as another control group.

### Characterization of hydrogels

FTIR spectra were acquired using a Nicolet 5,700 (Thermo Scientific) at room temperature from 4,000 to 400 cm^−1^. The morphology and surface elemental composition of the hydrogels were visualized under SEM (3400-N, Hitachi, Tokyo, Japan). The rheological behavior of the hydrogels was evaluated by a HAAKE MARS III rheometer. The pre-solution processability was tested under rotation ramp mode from 0.01 to 100 s^−1^ in 1 min at 37°C. Dynamic frequency sweep tests were carried out from 15 to 0.1 Hz at 37°C with an oscillatory strain of 1% at the thickness of 1 mm. The microstructure of the materials was examined by X-ray diffraction (XRD, Rigaku D/Max2550, Tokyo, Japan) with a scan range of 10 to 60^o^. The mechanical properties of hydrogels were evaluated by an electronic mechanical testing machine (SANS CMT2503, Guangzhou, China). Hydrogel samples were fabricated in a cylindrical shape (8 mm in diameter and 10 mm in height) and tested at a speed of 10 mm min^−1^. The swelling test was evaluated by gravimetric analysis. The original hydrogel was weighed, giving *W*_*o*_, and then hydrogels were immersed in phosphate-buffered saline (PBS). The hydrogels were taken out from PBS at different time intervals and weighed again, to find *W*_*s*_, until swelling equilibrium. The swelling ratio was then calculated according to the following equation:

$$\text{Swelling ratio}=\frac{W_{s}-W_{o}}{W_{o}}\times 100\%$$ (1)

The degradation of the samples was also recorded using gravimetric analysis. The prepared hydrogels were weighed to find *W*_*o*_ and then incubated in PBS with 2 CDU mL^−1^ collagenase type I solution at 37°C for 1 week. The hydrogels were weighed every day to find *Wt*. The degradation ratio was then calculated according to the following equation:

$$\text{Degradation ratio}=\frac{W_{0}-W_{t}}{W_{0}}\times 100\%$$ (2)

### CCK-8 cell proliferation assay

BMSCs were isolated from 3-week-old male Sprague-Dawley rats. The cells were seeded into 96-well plates at a density of 2 × 10^3^ cells per well and cultured in hydrogel-conditioned medium. On days 1 and 3, the conditioned medium was replaced, and 10% CCK-8 solution was added to each well. The plates were then incubated at 37°C for 1 h in a humidified incubator. After incubation, the absorbance at 450 nm was measured using a microplate reader.

### Cell culture and viability assessment

BMSCs were cultured in hydrogel-immersed conditioned medium. Cells were seeded at a density of 2 × 10^4^ cells per well in a 24-well plate, and 500 μL of conditioned medium was added. At 24 and 48 h, cell viability was assessed using a live/dead cell viability assay kit. The cells were subsequently imaged and analyzed using a confocal microscope.

### ALP and ARS assays

BMSCs were seeded into 24-well plates at a density of 1 × 10^4^ cells per well and cultured in conditioned medium for 3 and 7 days, with medium changes every 2 days. At each time point, the cells were harvested, washed three times with PBS, and fixed with 4% paraformaldehyde. The cells were then stained using an alkaline phosphatase staining kit and observed under a bright-field microscope. For Alizarin Red S (ARS) staining, the culture plates were pre-coated with 0.1% gelatin. BMSCs were seeded into 6-well plates at a density of 1 × 10^5^ cells per well and cultured in conditioned medium. At 14 and 21 days, the cells were stained with ARS solution to visualize mineralized nodules, and images were captured.

### Immunofluorescence assay for osteogenic marker proteins

Immunofluorescence staining was performed to evaluate the expression of osteogenic marker proteins. Glass coverslips were placed in 6-well plates, and 2 × 10^4^ cells were seeded onto each coverslip. Once the cells reached approximately 70% confluency, the medium was replaced with conditioned medium. The expression of Runx2 was assessed on day 7, while OCN and Col-1 expressions were evaluated on day 14. The coverslips were washed twice with PBS, followed by fixation with 4% paraformaldehyde for 30 min. After fixation, the cells were permeabilized with 0.2% Triton X-100. Blocking was carried out using a blocking solution containing 10% goat serum for 30 min. Primary antibodies, diluted in the blocking solution, were incubated with the coverslips overnight at 4°C. On the following day, the coverslips were incubated with secondary antibodies at room temperature for 2 h, followed by PBS washes. Actin filaments were stained with phalloidin, and the nuclei were counterstained with DAPI. Finally, the coverslips were mounted and observed under a confocal microscope.

### Osteogenic gene expression analysis

RT-qPCR was employed to evaluate the expression of osteogenic genes. BMSCs were seeded into 6-well plates at a density of 1 × 10^5^ cells per well and cultured until approximately 70% confluency. At this point, the medium was replaced with hydrogel-conditioned medium to promote osteogenic differentiation. After culturing for 7 and 14 days, total RNA was extracted using an RNA extraction kit according to the manufacturer’s instructions. The purity and concentration of the extracted RNA were measured using a spectrophotometer by assessing the OD260/OD280 ratio. Subsequently, 1 μg of total RNA was reverse transcribed into complementary DNA (cDNA) using a reverse transcription kit. RT-qPCR was performed using a SYBR Green PCR master mix on a real-time PCR detection system. Specific primers targeting osteogenic marker genes, including Runx2, OPN, OCN, and Col-1, were used, with primer sequences detailed in Supplementary Table S1. The amplification protocol included an initial denaturation at 95°C for 5 min, followed by 40 cycles of denaturation at 95°C for 10 s, annealing at 60°C for 30 s, and extension at 72°C for 30 s β-Actin was used as the internal reference gene. Relative gene expression levels were calculated using the 2^−ΔΔCT^ method, normalized to β-actin expression (Tan et al., 2022). All experiments were conducted in triplicate, and the results are presented as mean ± standard deviation (SD).

### *In vivo* cranial defect model and scaffold implantation

The rat cranial defect model was utilized to assess the osteogenic efficacy of the scaffold. All animal experimental procedures and care were approved by the Animal Ethics Committee of Xuzhou Medical University. Male Sprague-Dawley rats weighing approximately 250 g were used to establish a critical-size calvarial defect model. Rats were anesthetized via intraperitoneal injection of 1% sodium pentobarbital (40 mg/kg). Two 4 mm diameter cranial defects were created on each side of the skull using a microbone drill. Immediately after the bone was removed, the cranial defects were rinsed with saline solution, and the gel scaffolds were implanted. Twenty-four rats were randomly assigned to one of four groups: (1) control (no treatment for the cranial defects), (2) GelMA scaffolds, (3) GK scaffolds, and (4) GKP scaffolds. After 4 and 8 weeks of implantation, Euthanasia of rats was performed by intraperitoneal injection of 200 mg/kg sodium pentobarbital solution (3%), and their skulls were collected for micro-CT analysis and histological assays.

#### Microcomputed tomography analysis

Micro-CT (Bruker, Belgium) was employed to assess bone regeneration in the four groups. Skulls were harvested and fixed in 4% paraformaldehyde for 24 h prior to CT scanning. Samples were scanned at an 18 μm pixel resolution (65 kV, 380 μA). The defect region was visualized in the coronal, sagittal, and transaxial planes, using skull tissue as a reference, with the Dataviewer software. A circular region of interest (ROI) with a 4-mm diameter corresponding to the defect site was selected. CTAn software was used to analyze the regenerated neo-tissue, with parameters including BV, BV/TV, Tb. Th, Tb. N, and Tb. Sp. Representative 2D and 3D reconstruction images were generated using Dataviewer and CTVol software, respectively.

#### Histological and immunohistochemical evaluation

For bone histology evaluation, samples were fixed in 4% paraformaldehyde for 24 h and then decalcified in 15% ethylenediamine-tetraacetic acid (EDTA) for 2 weeks. After decalcification, the samples were dehydrated using a gradient of alcohols, embedded in paraffin, and sliced into 5-μm thick contiguous sections for H&E, MT, and immunofluorescence analysis. Immunofluorescence staining was performed using antibodies against OCN and Runx2. The sections were subsequently imaged using a confocal fluorescence microscope.

### Statistical analysis

All data are presented as the mean ± SD. Statistical analysis was assessed using GraphPad Prism 10.0 statistical software. All quantifications were analyzed by ImageJ software. Two-way analysis of variance was used to conduct the statistical analysis, and p < 0.05 was considered statistically significant.

## Data Availability

The original contributions presented in the study are included in the article/Supplementary Material, further inquiries can be directed to the corresponding authors.
