# Supplementary material for: Injectable organic-inorganic hybrid hydrogels for bone defect repair
Source: Front Bioeng Biotechnol. 2025 Mar 18;13:1563546. doi: 10.3389/fbioe.2025.1563546 (PMC11959084; doi:10.3389/fbioe.2025.1563546)
Supplement: Supplementary file 1 [file DataSheet1.docx]

**Support Information**

**Injectable Organic-Inorganic Hybrid Hydrogels for Bone Defect Repair**

Huan Zhang,^1, 2, 3, 4 †^ Shuo Ding,^5 †^ Huai Xue,^2, 3, 4^ Shuguang Wang,^3, 4^ Xiaoyu Quan,^3, 4^ Dong Zhang,^6 *^ Xiao Liu,^3, 4 *^ and Hai Tang ^1 *^

^1^ Department of Orthopaedics, Beijing Friendship Hospital, Capital Medical University, No. 95, Yong An Road, Xicheng District, Beijing 100050, People’s Republic of China.;

^2^ School of Second Clinical Medicine, Xuzhou Medical University, Xuzhou, Jiangsu, 221002, China.

^3^ Department of Trauma Center, The Affiliated Hospital of Xuzhou Medical University, Xuzhou, Jiangsu, 221002, China.

^4^ Department of Emergency Medicine, The Affiliated Hospital of Xuzhou Medical University, Xuzhou, Jiangsu, 221002, China.

^5^ Department of Emergency, Fengxian People’s Hospital, Xuzhou, Jiangsu, 221700, China.

^6^ The Wallace H. Coulter Department of Biomedical Engineering, Georgia Institute of Technology and Emory University, Atlanta, GA 30332, USA.

**^*^ Correspondence:**

Dong Zhang, dzhang470@gatech.edu

Xiao Liu, docliuxiao@163.com

Hai Tang, tanghai@ccmu.edu.cn

^†^ H. Z. and S. D. contributed equally to this work. ^†^ H. Z. and S. D. contributed equally to this work.


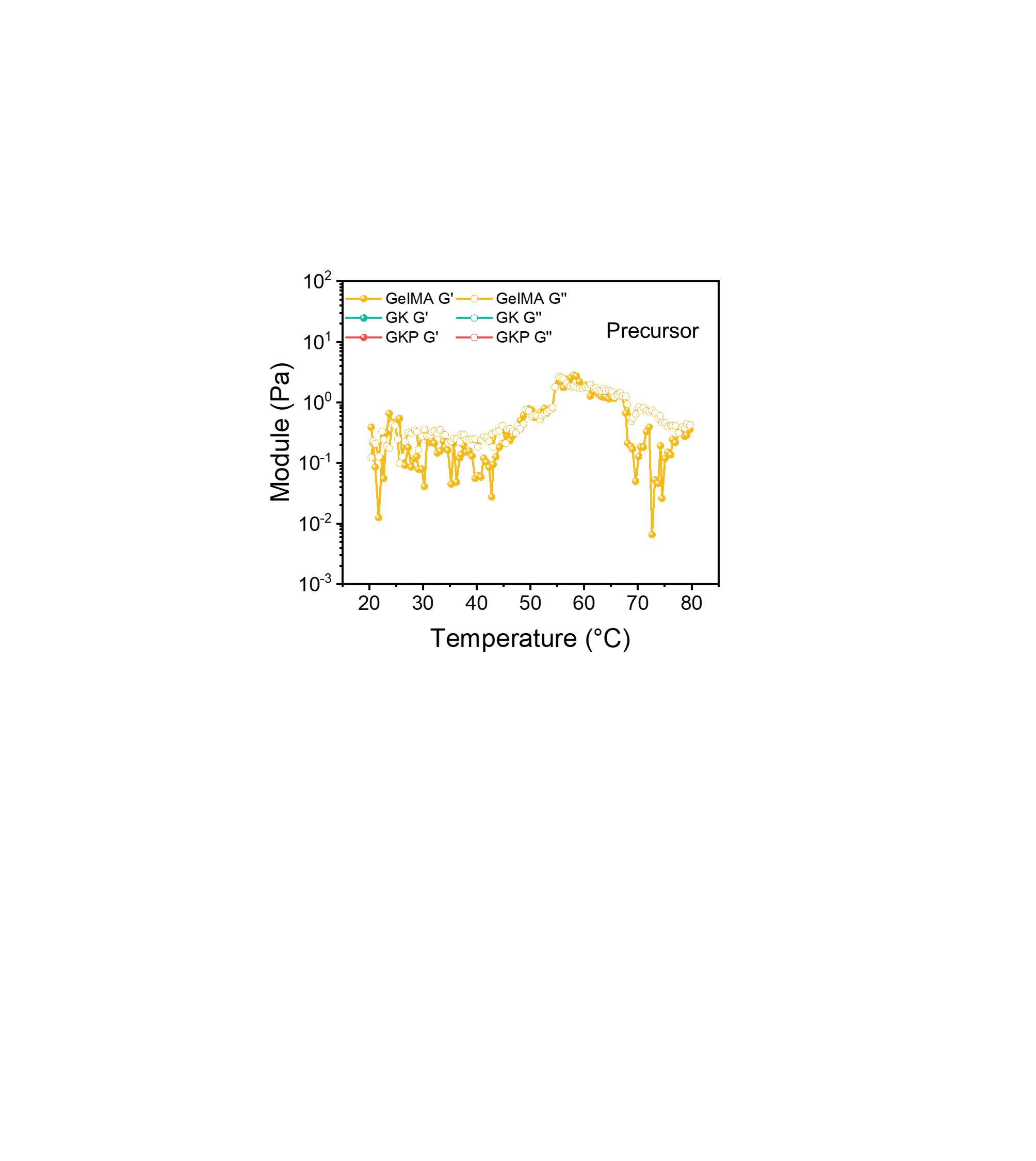


**Figure S1.** Modulus variation of precursor solutions at different temperatures.


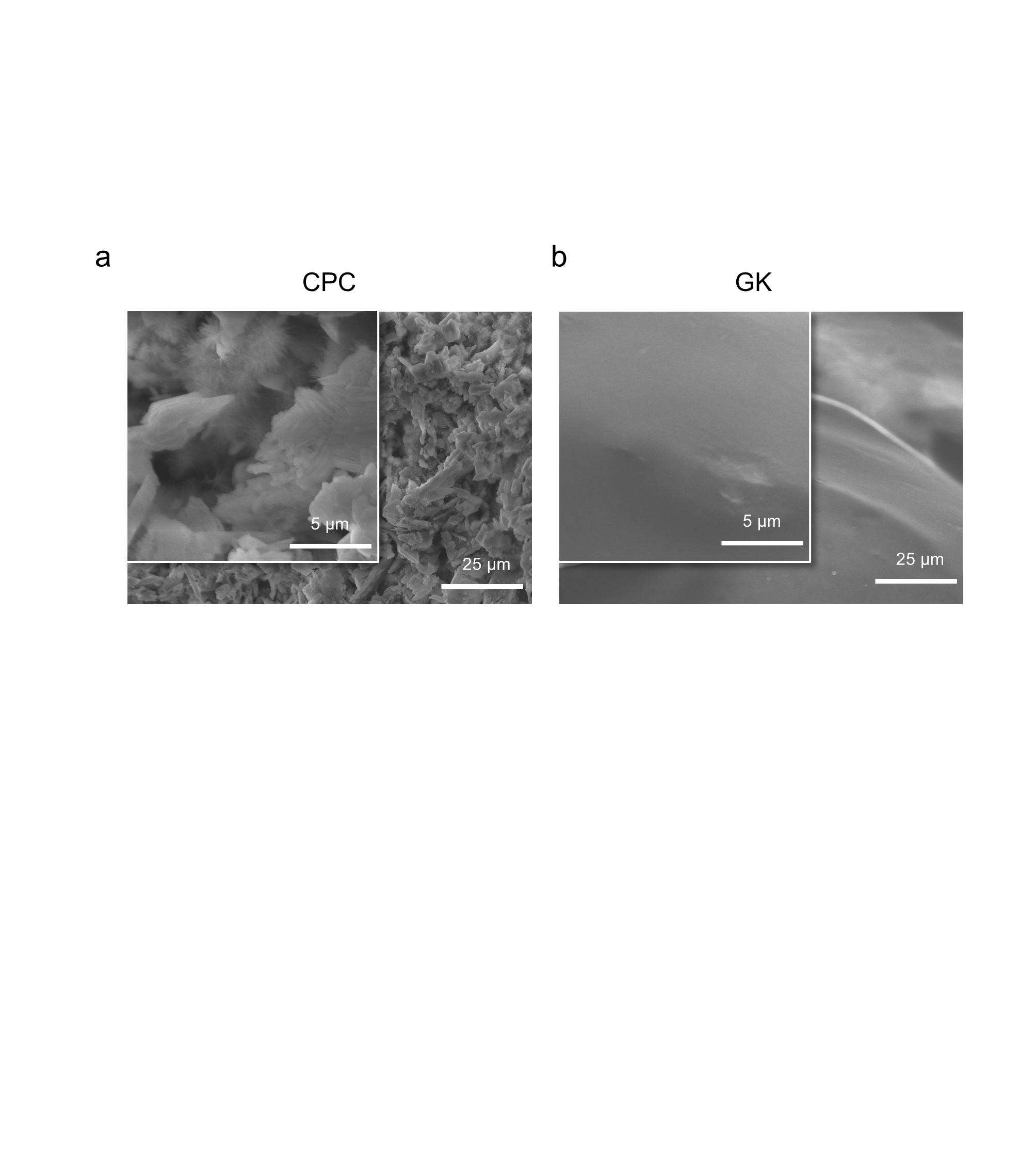


**Figure S2.** SEM image of CPC and GK.


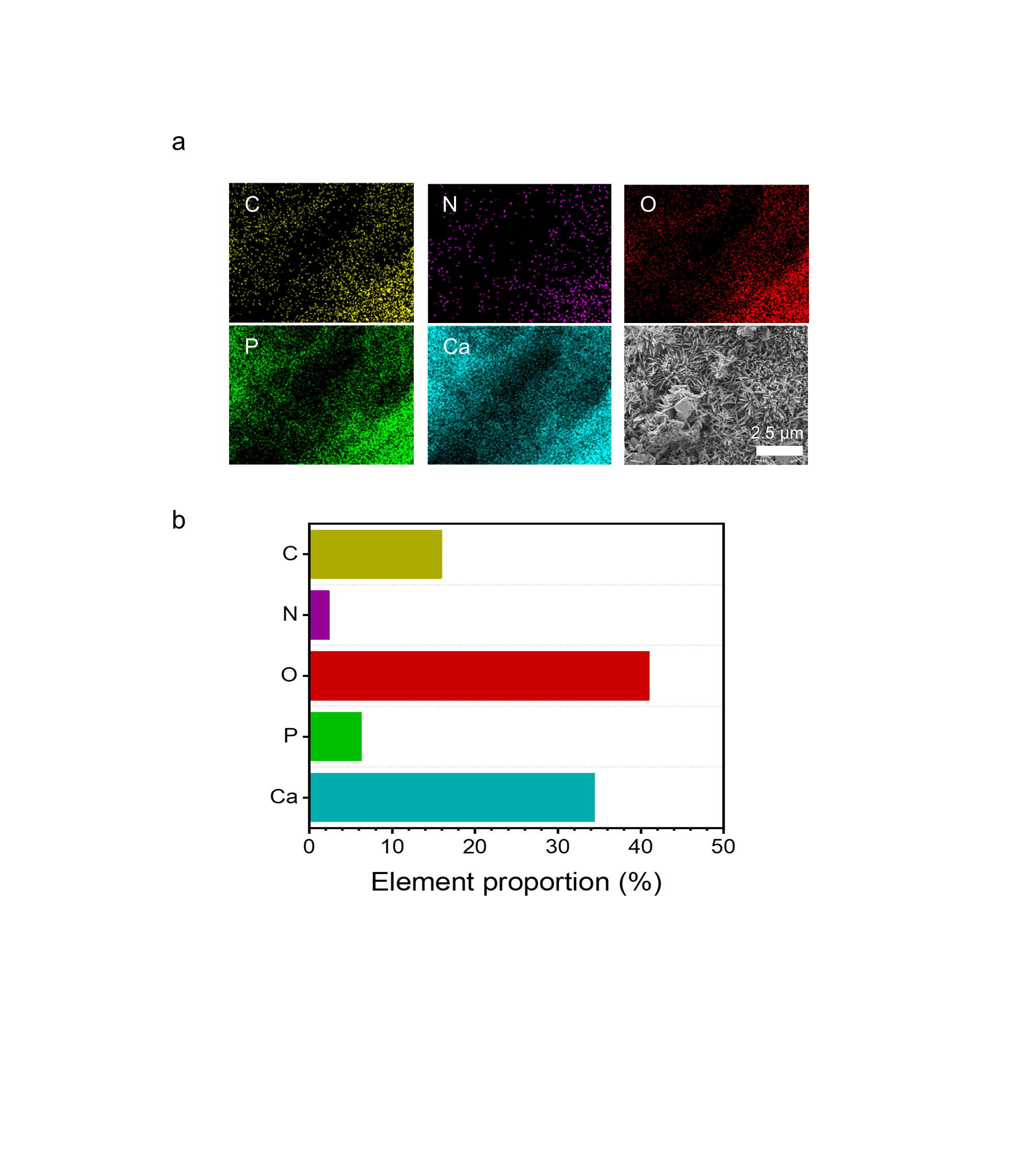


**Figure S3.** The image and elemental proportion and distribution of GKP.


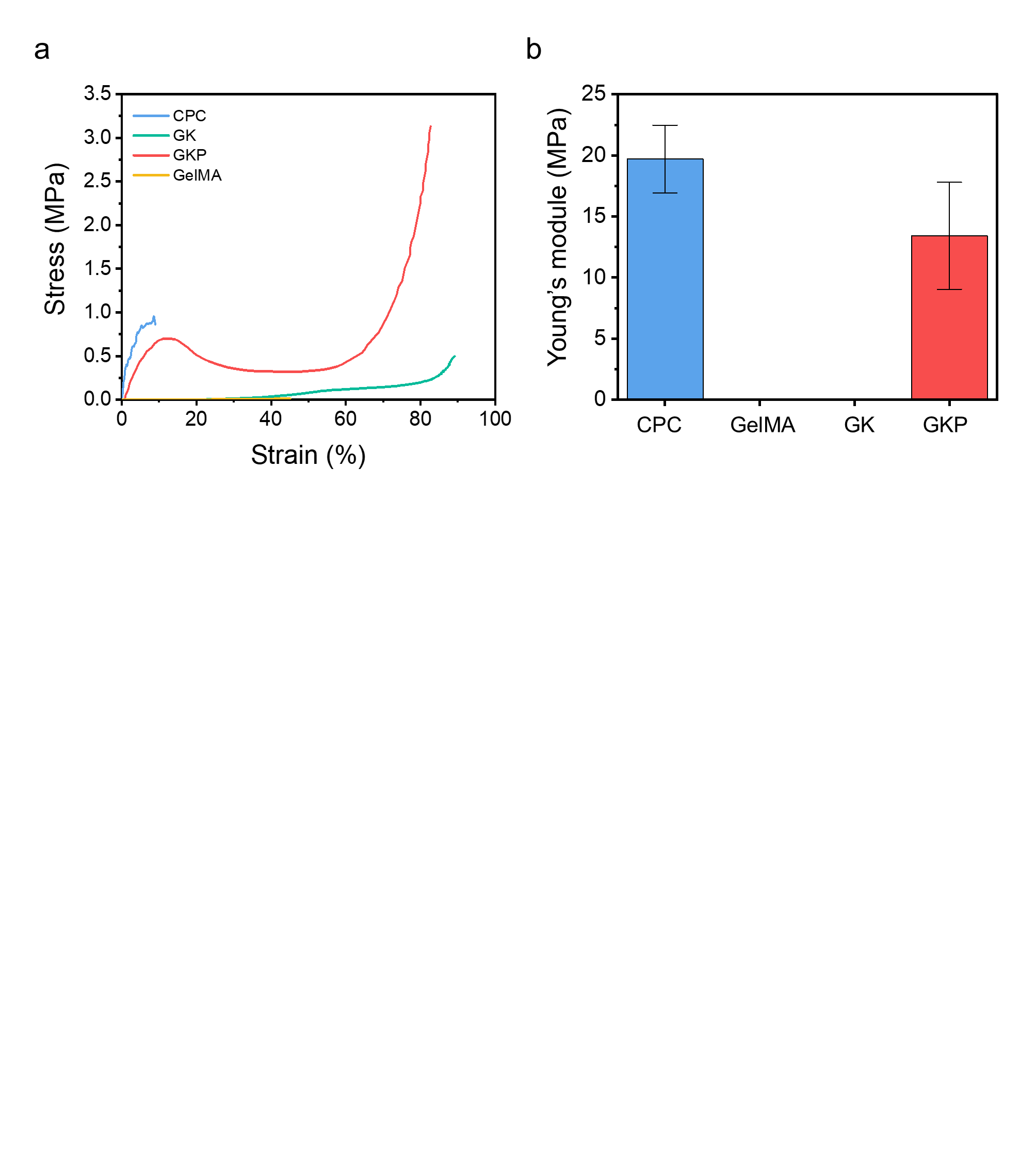


**Figure S4.** a) Typical compressive strain curves of different samples and (b) Young’s modulus of different hydrogels.


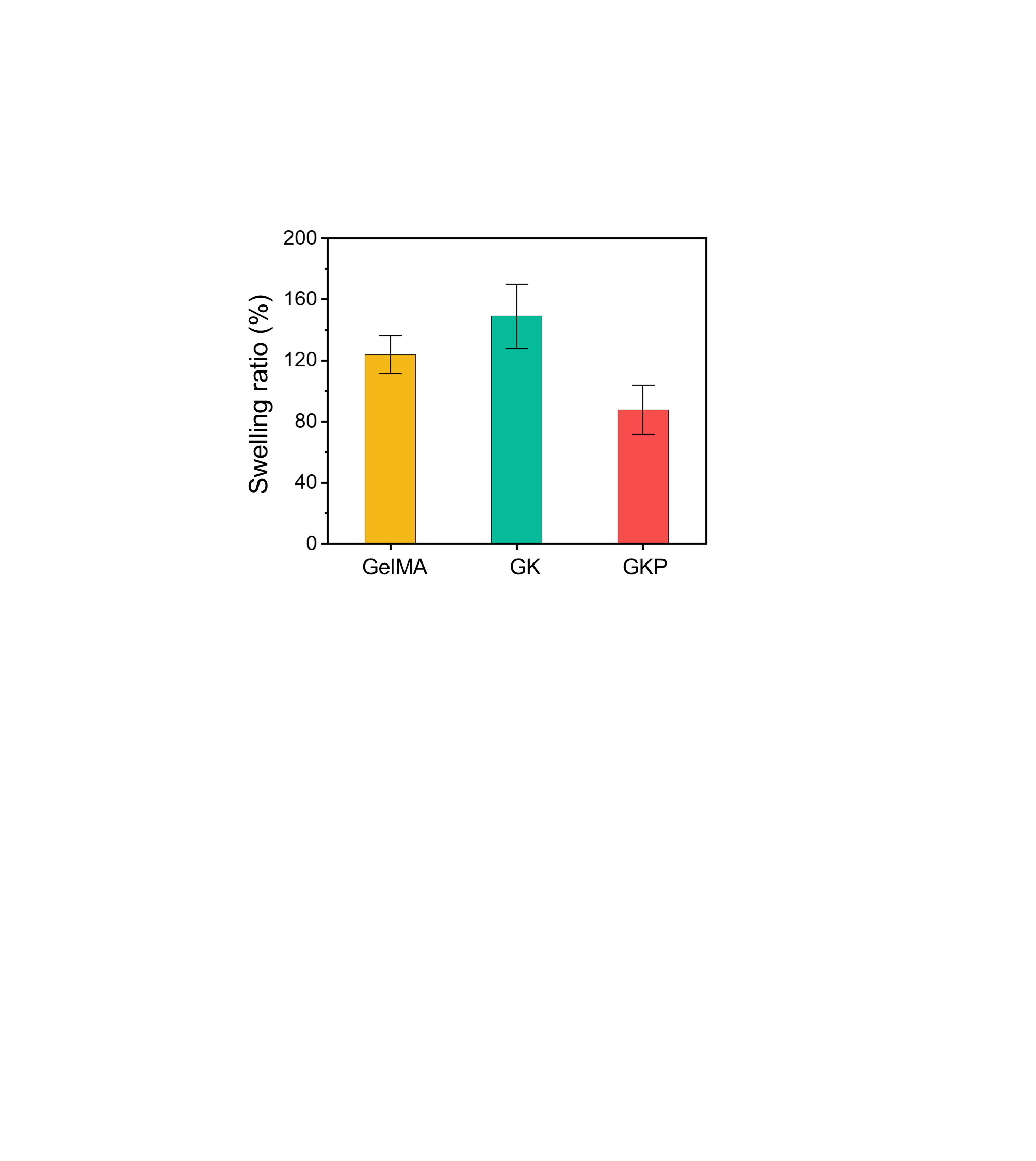


**Figure S5.** Swelling properties of different hydrogels.


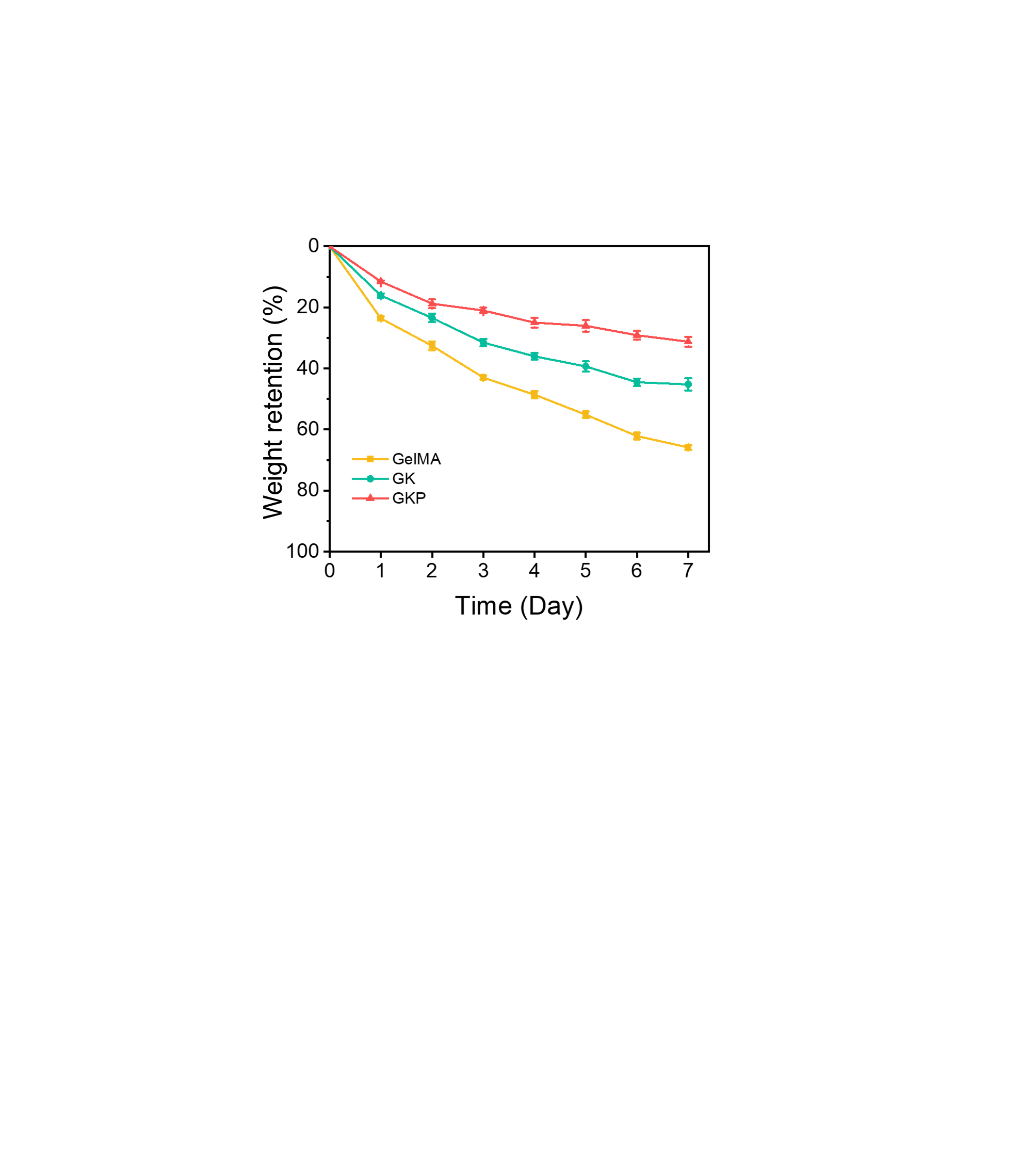


**Figure S6.** Biodegradation behavior of hydrogels.

**Figure S7.** CCK-8 assay showing BMSCs proliferation at 1 and 3 days.

**Table S1.** list of RT-qPCR primers used in this study.

| **Gene** | **Prime sequence (F, forward; R, reverse)** |
| --- | --- |
| β-Actin | F: GTCCCTCACCCTCCCAAAAG R: GCTGCCTCAACACCTCAACCC |
| Runx2 | F: GACTGTGGTTACCGTCATGGC  R: ACTTGGTTTTTCATAACAGCGGA |
| OCN | F: GGACCATCTTTCTGCTCACTCTGC  R: TGTTCACTACCTTATTGCCCTCCTG |
| OPN | F: TGATGAATCCGATGAACTGG  R: AGTGATGTGAAGTCCTCCTCTGT |
| Col-1 | F: ACTGGCAACCTCAAGAAGTCCC  R: AAGTTCCGGTGTGACTCGTGC |


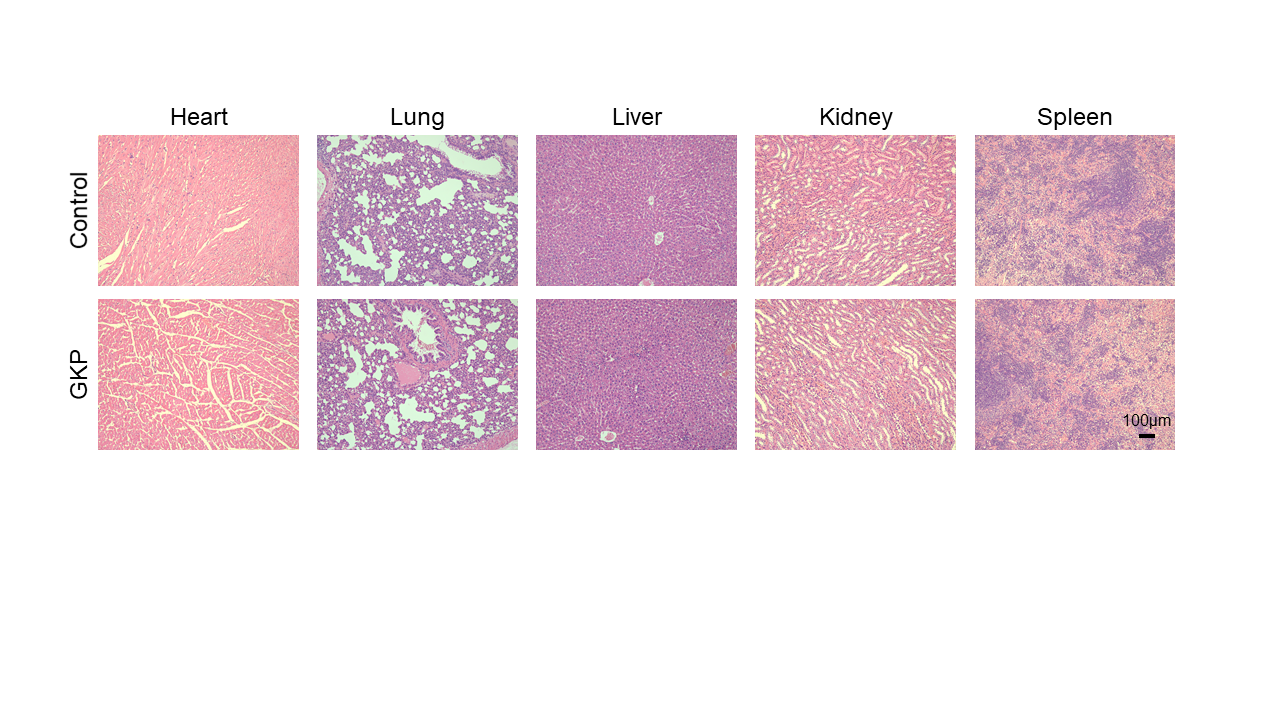


**Figure S8.** HE staining images of heart, lung, liver, kidney and spleen from rats at 8 weeks post-surgery.
